# Dense seismic arrays deny a massive magma chamber beneath the Taipei metropolis, Taiwan

**DOI:** 10.1038/s41598-020-80051-4

**Published:** 2021-01-13

**Authors:** Yu-Lien Yeh, Wei-Hau Wang, Strong Wen

**Affiliations:** Department of Earth and Environmental Sciences, National Chung Cheng University, Minxiong, Taiwan, ROC

**Keywords:** Seismology, Volcanology, Natural hazards

## Abstract

Several recent studies suggest that the Tatun Volcano Group (TVG) in the Taipei metropolis of Taiwan is still active with a mappable magma chamber beneath it. Here we report new seismic evidence from dense seismic arrays in northern Taiwan to refute the presence of a massive magma chamber. We investigated two near Taipei earthquakes with focal depths of ~ 140 km. We found that all the waveforms exhibited distinct S waves even when they traversed across the previously postulated magma chamber. Instead, the S-wave shadows found in the previous study may result from seismic waves traveling through a magma diapir above the subducting Philippine Sea Plate offshore northern Taiwan. Moreover, we found the P-wave delay increased with hypocentral distance when the seismic waves propagated through the footwall (west side) of the Shanchiao fault, regardless of whether they traversed across the postulated magma chamber. Our study results also indicate no abnormal attenuation when seismic rays traversed across the postulated magma chamber. Furthermore, the average ${Q}_{P}/{Q}_{S}$ ratio around the TVG is less than 1, which implies that scattering attenuation is dominant. We conclude that a highly fractured rock body is beneath the TVG with a tiny fraction of magma instead of a massive magma chamber. Without sufficient magma supply, the TVG may stay dormant (except for small phreatic eruptions).

## Introduction

**Figure 1:**
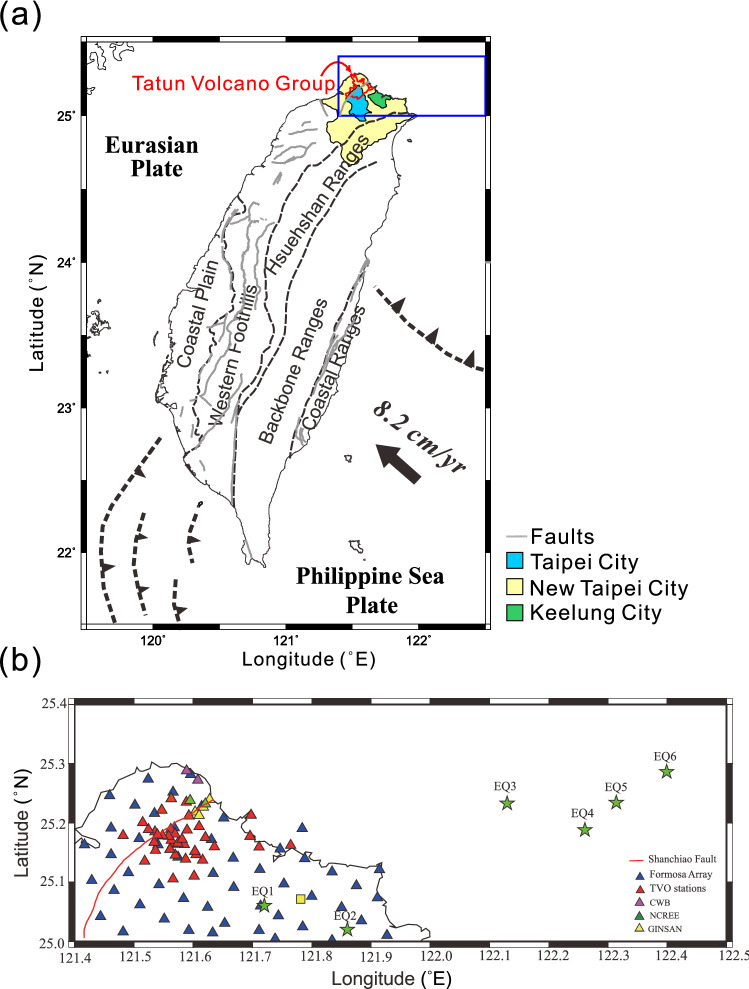
**(a)** Tectonic setting and the location of the Tatun Volcanic Group (TVG). **(b)** The distribution of the seismic stations employed in this study. The black stars denote the epicenters of the six earthquakes (EQ1–EQ6) adopted in this research. The yellow square denotes the reference station NWF. This figure is generated by GMT 6.0.0 http://gmt.soest.hawaii.edu/projects/gmt and CorelDRAW 2020 https://www.coreldraw.com/tw/?link=wm.

The Tatun Volcano Group, comprising ~ 20 volcanoes, is situated in the Taipei metropolis of Taiwan above the western boundary of the subducting Philippine Sea Plate (Fig. 1a). The TVG is cut in the middle by the Shanchiao fault (Fig. 1b), an eastward-dipping, listric normal fault^1^. Early studies indicated that the TVG had been active during 2.8–0.1 Ma^2–4^, but has stayed dormant ever since^5^. Recent discoveries, however, challenge this viewpoint. The new findings include a small body (ca. 0.05 km^3^) of 6 Ka debris flow that contains pyroclastic fragments at Mt. Cising^6^, the 1367-year-old magnetites in a piece of volcanic rock at Mt. Shamao^7^, and the presence of abnormally high ^3^He/^4^He volcanic gases^8,9^. All these new findings imply that the TVG is being fed by an active magma chamber.

However, such a magma chamber had never been identified by seismic tomographic studies until a recent report by Lin^10^, who declared that a massive magma chamber with a size of $350~{\rm{km}}^{3}$ to $936~{\rm{km}}^{3}$ is embedded right beneath the Taipei metropolis at depths of about 28–34 km after studying four distant subduction events. He found S waves vanished and P waves lagged as the seismic waves of these events propagated through the postulated magma chamber. He further estimated that the P-wave velocity might drop up to 40% inside the magma reservoir. Although these two lines of evidence seem convincing, the events (EQ3-EQ6, Fig. 1b) that Lin adopted are too far and too deep (over 200 km) from the TVG to pinpoint the location of the attenuation source for S-wave shadows and P-wave delays without presuming its depth. To validate the postulated magma chamber under the Taipei metropolis, we investigated the waveforms, ray paths, and the differential ${t}_{P}^{*}$ and ${t}_{S}^{*}$ of two recent subduction earthquakes (EQ1 and EQ2, Fig. 1b) that occurred under the Taipei metropolis. We aimed to shed light on the origin of the S-wave shadows and better understand the physical properties beneath the TVG.

## Data and methods

Figure 1 shows our study area and the seismic stations selected from five seismic networks: the Taiwan Volcano Observatory at Tatun (TVO), the NCREE Array, the Ginsan Array, the Formosa Array (FA), and the CWB Array. Six earthquakes (EQ1-EQ6) were employed in this study with their origin times, locations, focal depths, and magnitudes listed in the Supplementary Table S1. It is worth noting that the FA has been operating since April 1, 2018. As a result, this array only recorded the waveforms of EQ2.

We have investigated the seismic waveforms of EQ1 and EQ2. Both events nucleated on top of the subducting Philippine Sea Plate with focal depths of 138 km and 140 km. The epicentral distances from these events to the postulated magma chamber are less than 25 km. The unique foci of these two events allow us to investigate the physical properties beneath the TVG with a closer look via seismic waveforms, arrival times, and attenuation characteristics.

We used the vertical and transverse components of the seismic waveforms radiating from these two events to identify their P and S waves. We then calculated the ray paths from each earthquake by employing a 3D ray-tracing technique^11^ with the seismic velocity structure derived by Huang et al*.*^12^. Thereby, we could recognize which seismic waves passed through the postulated magma chamber. Estimating the δt* for both P and S waves is crucial for identifying the attenuation sources and justifying if the attenuation is intrinsic or scattering. According to Sherbaum^13^, the observed spectrum at the station j for the event i can be expressed as

1 $${\rm{A}}_{ij}\left(\rm{f}\right)={\rm{S}}_{ij}(\rm{f})\cdot {\rm{I}}_{j}(\rm{f})\cdot {\rm{R}}_{j}(\rm{f})\cdot {\rm{B}}_{ij}(\rm{f}),$$

where f is the frequency, ${\rm{S}}_{ij}(\rm{f})$ the source spectrum, ${\rm{I}}_{j}(\rm{f})$ the instrument spectrum, ${\rm{R}}_{j}(\rm{f})$ the site response, and ${\rm{B}}_{ij}(\rm{f})$ the absorption along the ray path between the event $i$ and the station $j$. The source spectrum can be expressed as

2 $${\rm{S}}_{ij}\left(\rm{f}\right)=\frac{{M}_{0}P({\theta }_{ij},{\varnothing }_{ij})}{4\pi \rho {d}_{ij}{v}^{3}}\cdot \frac{{f}_{c}^{\gamma }}{{f}_{c}^{\gamma }+{f}^{\gamma }}=\frac{{\Omega }_{0}}{{d}_{ij}}\cdot \frac{{f}_{c}^{\gamma }}{{f}_{c}^{\gamma }+{f}^{\gamma }},$$

where ${M}_{0}$ is the seismic moment, P the radiation pattern, $\rho$ the density, ${d}_{ij}$ the hypocentral distance, $v$ the wave velocity, ${f}_{c}$ the corner frequency, and $\upgamma$ a constant. The variation of P would be small for a distant earthquake observed by a dense array such as this study. Hence, the ${\Omega }_{0}$ can be regarded as a constant. According to Rietbrock^14^,

3 $$ B_{i} j(f) = exp( - \pi ft_{ij}^{*} ),~and $$

4 $$ R_{j} (f) = exp( - \pi ft_{station}^{*} ), $$

where ${t}_{ij}^{*}$ is the whole path attenuation operator, and ${t}_{station}^{*}$ is the local site amplification operator,

Since the influence of instrument response is trivial by choosing a proper frequency band, Eq. (1) can be rewritten as

5 $${\rm{A}}_{ij}\left(\rm{f}\right)=\frac{{\Omega }_{0}}{{d}_{ij}}\cdot \frac{{f}_{c}^{\gamma }}{{f}_{c}^{\gamma }+{f}^{\gamma }}\cdot{ \rm{ exp}}(-\pi f{(t}_{ij}^{*}+{t}_{station}^{*})),~or$$

6 $${t}_{ij}^{*}+{t}_{station}^{*}=\frac{{\text{ln}}({\text{A}}_{ij}({\text{f}})\cdot {d}_{ij})+ {\text{ln}}\frac{{f}_{c}^{\gamma}+{f}^{\gamma}}{{\Omega}_{0}{f}_{c}^{\gamma}}}{-\pi f}$$

The relationship between ${t}_{ij}^{*}$ and the quality factor Q is

7 $${t}_{ij}^{*}={\int }_{path}1/(Q\cdot v) ds.$$

To eliminate the source effect, from Eq. (6), we can calculate relative ${t}_{ij}^{*}$ for the station $j$ with respect to ${t}_{ik}^{*}$ for the station $k$, such that

8 $$\updelta {t}_{ij,k}^{*}={t}_{ij}^{*}-{t}_{ik}^{*}\approx \frac{{\rm{ln}}\left(\frac{{A}_{ik}(f)}{{A}_{ij}(f)}\right)+{\rm{ln}}\left(\frac{{d}_{ik}}{{d}_{ij}}\right)}{\pi f}.$$

**Figure 2:**
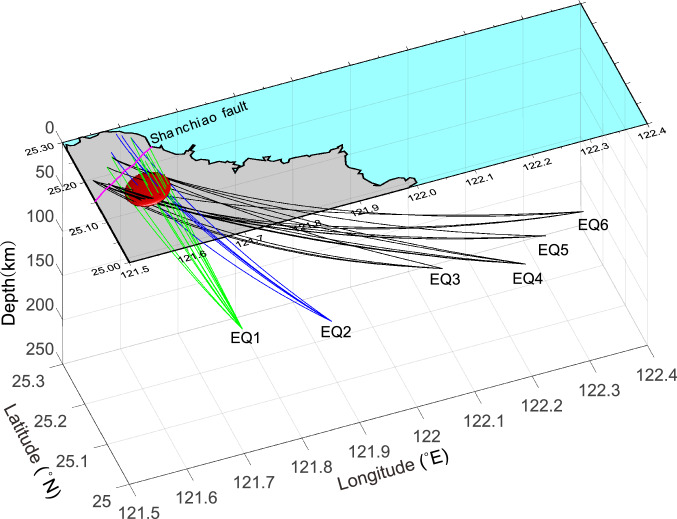
A diagram illustrating all the seismic rays traversing across the postulated magma chamber (the red ellipsoid) at depths of 30 ± 3.3 km from the foci of the EQ1 to EQ6. This figure is generated by MATLAB 2020a https://www.mathworks.com/products/matlab.html and CorelDRAW 2020 https://www.coreldraw.com/tw/?link=wm.

Note that Eq. (8) is under the assumption that the variation of ${t}_{station}^{*}$ is much smaller than that of $\updelta {t}_{ij,k}^{*}$. In this study, we take the station NWF (Fig. 2) established by the Central Weather Bureau of Taiwan as the reference station. Because the station NWF is adjacent to the epicenters of EQ1 and EQ2 and not too far away from the other observation stations, employing $\updelta {t}_{ij,k}^{*}$ will reduce the path effect near the source area. Also, note that $\updelta {t}_{ij,k}^{*}$ is a function of the frequency. In this study, we adopted the average $\updelta {t}_{ij,k}^{*}$ in the frequency band of 2–4 Hz, because this frequency band contains the dominant seismic energy of the studied body waves, i.e., higher S/N ratios.

The velocity spectrum can be obtained by applying the Fast Fourier Transform and the multitaper filter of Thomson^15^ to the waveforms. The latter will make the velocity spectrum smoother. We calculated $\updelta {t}_{ij,k}^{*}$ for both P and S waves (hereafter referred to as $\updelta {t}_{P}^{*}$ and $\updelta {t}_{S}^{*})$ by applying the abovementioned procedure to the waveforms in a time window of 2.56 seconds^16^ after the first arrivals of either P or S waves. In so doing, we could reduce contaminations in our data by other seismic phases.

## Results

### Origin of the S-wave shadows

**Figure 3:**
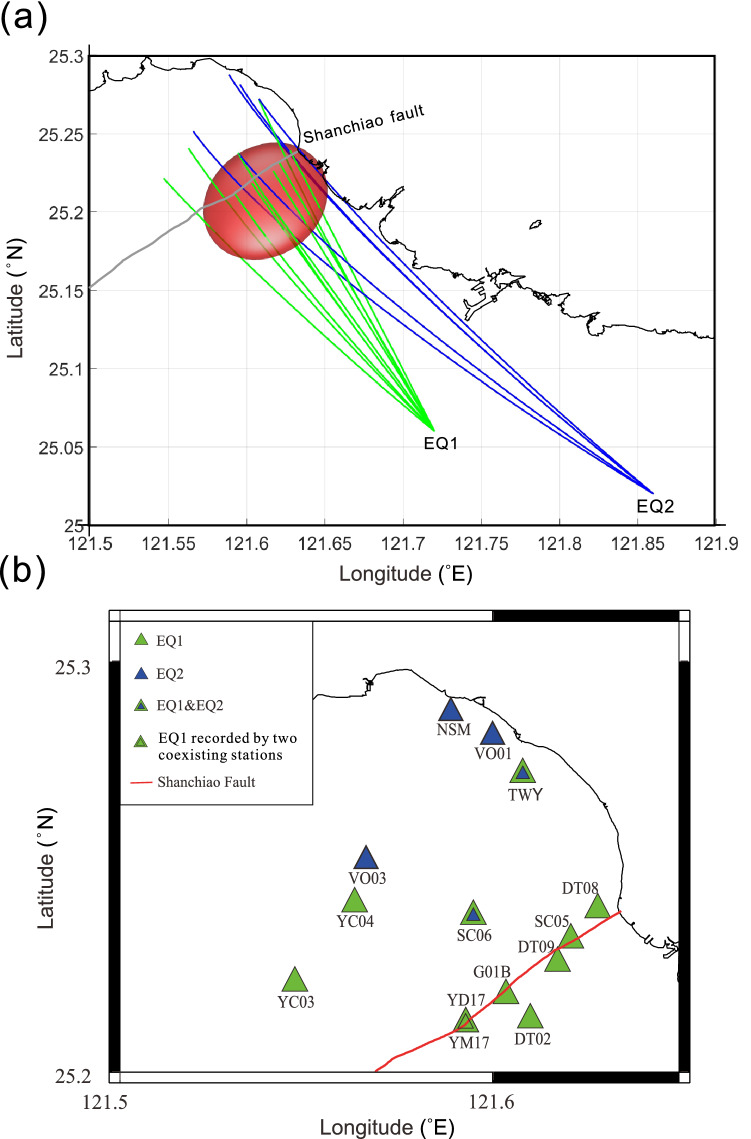
**(a)** The map view of the seismic rays passing through the postulated magma chamber from the EQ1 and EQ2. **(b)** The seismic stations receiving the seismic rays shown in **(a)**. The green triangles denote the seismic stations that recorded EQ1, and the blue triangles denote the seismic stations that recorded EQ2. The blue triangles with green borders denote the stations recorded both EQ1 and EQ2, and the green triangle with double borders denote there are two seismometers at the same site and both of them recorded EQ1. Note that stations VO01 and VO02 were established after the EQ1.

**Figure 4:**
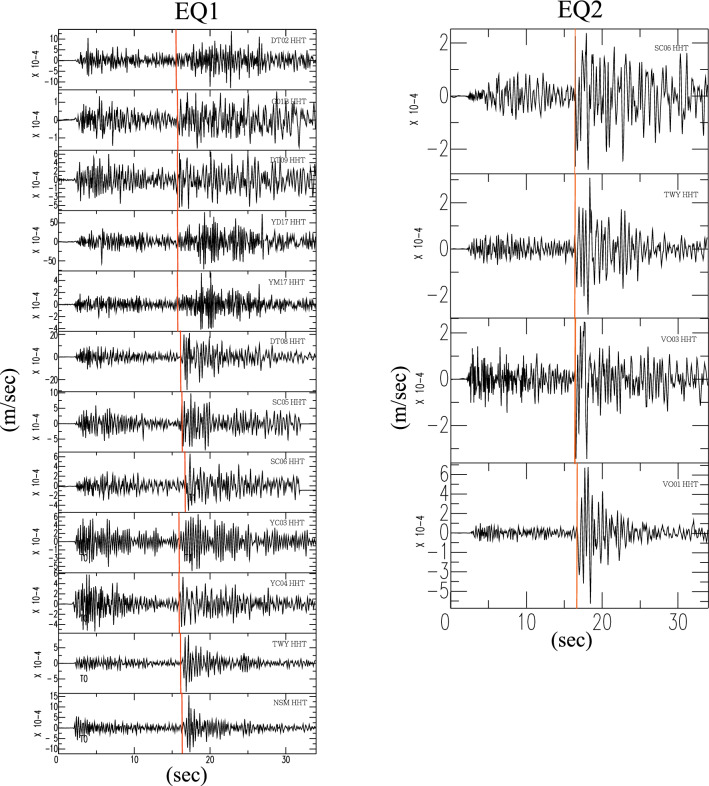
The transverse components of the seismic seismograms corresponding to the seismic rays shown in Fig. 3 with their first P-wave arrivals aligned. Note that all the waveforms exhibit distinct S waves with their first arrivals marked by red lines.

We re-established the shape of the postulated magma chamber by assuming it is an ellipsoid, which resides in depths of 30 $\pm$ 3.3 km. We searched for the minimum-volume magma chamber that just encircles all the seismic rays with S-wave shadows reported by Lin^10^ (EQ3-EQ6). Figure 2 shows the optimal magma chamber and all the seismic rays which traversed across the chamber from EQ1 to EQ6. To test if the S-wave shadows originated from the postulated magma chamber, we examined the waveforms of 16 seismic rays that passed through the postulated magma chamber from EQ1 and EQ2 (Fig. 3). Unlike the previous observations, we found that all these waveforms exhibit distinct S waves (Fig. 4) except at YM17, YD17, and DT02. The S waves observed in these nearby stations are recognizable but attenuated. We suspect they are influenced by a shallow hydrothermal reservoir and will discuss this issue in the latter section. We also investigated other waveforms of these two earthquakes with the recording stations shown in Fig. 1, but still failed to find any S-wave shadow (see Supplementary Figures S2 & S3).

**Figure 5:**
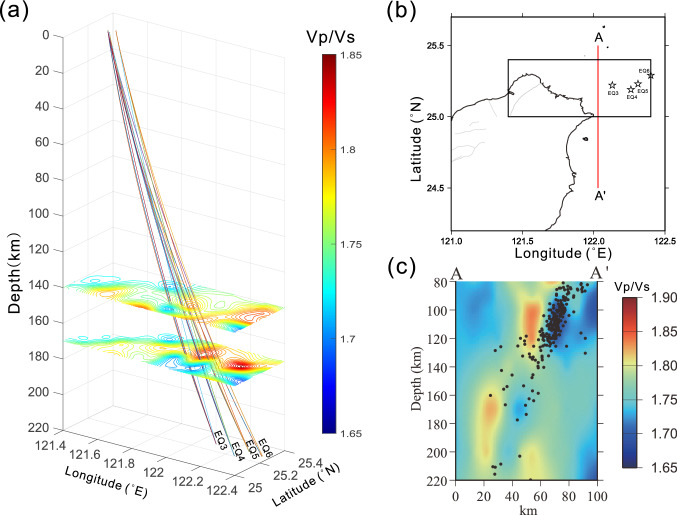
**(a)** The Vp/Vs structures (derived from Huang et al.^12^) overlapping with the ray paths across the postulated magma chamber from the EQ3-EQ6 shown in Fig. 1. Note all the rays passed through the regions with high Vp/Vs ratios at depths of 140 and 170 km offshore northeastern Taiwan. **(b)** The location of the profile AA’ with the rectangle showing the study area. **(c)** The cross-sectional diagram of AA’ showing two high-Vp/Vs diapirs above the subduction zone. The ray paths shown in **(a)** traversed across the deeper diapir (ca. 30 km south to point A). The black dots denote earthquakes.

Our findings reject the hypothesis of the presence of a massive chamber beneath the Taipei metropolis and northern Taiwan. If so, what is the origin of the S-wave shadows observed in the previous study? One clue is that all the earthquakes (EQ3-EQ6) without S waves shared common characteristics: they are offshore subduction events with focal depths over 200 km^10^. With these depths, one would expect the seismic waves might have a chance to travel through magma diapirs due to dehydration melting above the subduction zone. If so, S-wave shadows would form the area within or nearby magma diapir will become anelastic and rapidly loses shear rigidity as the homologous temperature approaches to one^17,18^. Such a magma diapir often exhibits a high-${V}_{P}/{V}_{S}$ ratio and a cigar-like body over the subducting plate. In fact, several previous studies^19–21^ have found this type of magma diapirs in offshore northern Taiwan. To test the possibility of this scenario, we overlapped the ray paths associated with S-wave shadows with the ${V}_{P}/{V}_{S}$ structure^12^ in northern Taiwan and discovered that all the ray paths traveled through the high-${V}_{P}/{V}_{S}$ regions between the depths of 140 and 170 km offshore northern Taiwan (Fig. 5). In the cross-section view (Fig. 5c), it appears that the magma diapir generates from the 220-km-deep subduction zone. There is another shallower magma diapir originating at a depth of 140 km, a likely magma source feeding the magma reservoir beneath Turtle Island offshore northeastern Taiwan. This finding accounts for the origin of the formation of S-wave shadows and explains why the subduction events with their epicenters on land or focal depths of less than 100 km offshore fail to exhibit S-wave shadows in the TVG.

### Attenuation in the TVG

**Figure 6:**
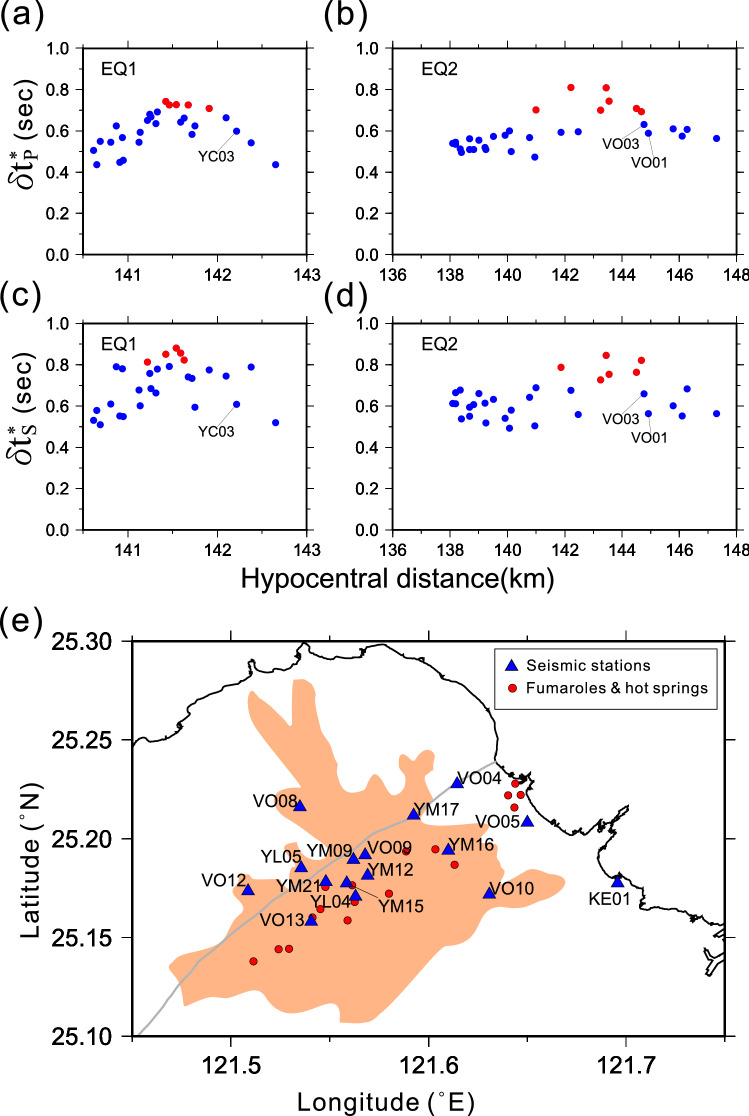
Diagram **(a–d)** illustrate the distributions of ${\delta t}_{P}^{*}$ and ${\delta t}_{S}^{*}$ against hypocentral distance for the EQ1and EQ2. The red dots denote the abnormal ${\delta t}^{*}$ that exceeds one standard deviation above the mean. Diagram **(e)** depicts the seismic stations (the blue triangles) with abnormal ${\delta t}^{*}$ overlapping with the geothermal activities (the red dots) and the Shanchiao fault (the gray line). The TVG is the area in brown.

Figure 6 illustrates the variations of $\updelta {t}_{P}^{*}$ and $\updelta {t}_{S}^{*}$ against the hypocentral distances for EQ1 (observed by the TVO array) and EQ2 (by the Formosa array). The red dots denote the $\updelta {t}_{P}^{*}$ or $\updelta {t}_{S}^{*}$ with their values greater than one standard deviation above the mean. The seismic stations associated with the anomalous $\updelta {t}_{P}^{*}$ or $\updelta {t}_{S}^{*}$ are plotted in Fig. 6e. We found that most of these stations (including stations YM17 and YD17) were closely associated with either the Shanchiao fault or the geothermal activities in the TVG, including hot springs and fumaroles. This finding implies the attenuation in the TVG might result from shallow fracture conduits or the hydrothermal reservoir below them. Such a shallow hydrothermal reservoir and conduits with depths above 3 km have been confirmed by low Rayleigh wave phase velocity^22^, anomalous P-wave delays^23^, and clusters of seismicity^24^. In contrast, further westward away from the geothermal field and the Shanchiao fault, no anomalous $\updelta {t}_{P}^{*}$ or $\updelta {t}_{S}^{*}$ can be found, including at the stations YC03 (Figs. 3, 6a,c), VO01, and VO03 (Figs. 3, 6b,d). Note that these three stations have recorded seismic waves traversing across the postulated magma chamber (Fig. 3).

**Figure 7:**
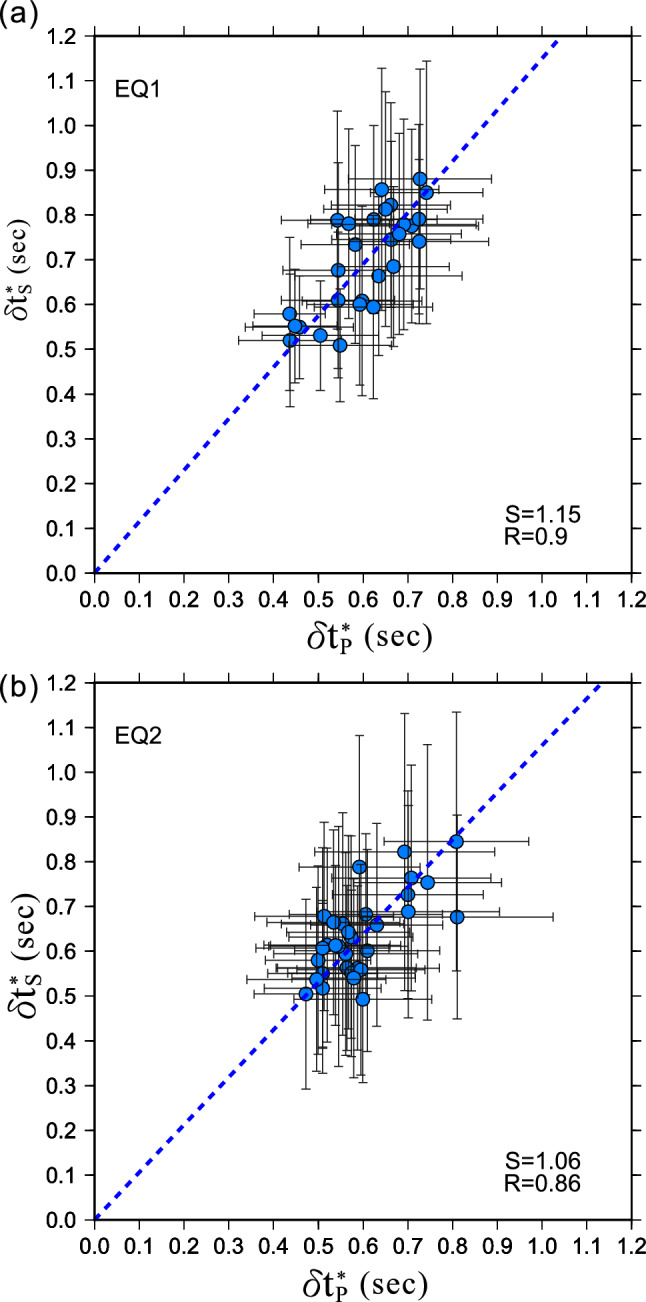
The plots of ${\delta t}_{S}^{*}$ against ${\delta t}_{P}^{*}$ for the event **(a)** EQ1and **(b)** EQ2. R denotes the correlation coefficient, S denotes the slope, and the error bars stand for one standard deviation.

In addition to the above observations, we also find that $\updelta {t}_{S}^{*}$ is linearly proportional to $\updelta {t}_{P}^{*}$ for both events (Fig. 7). For EQ1, the $\updelta {t}_{S}^{*}/\updelta {t}_{P}^{*}$ ratio is 1.15 $\pm$ 0.13, and EQ2 is 1.06 $\pm$ 0.12. According to Eq. (7), we can estimate $\frac{{Q}_{P}}{{Q}_{S}}\approx \frac{{\delta t}_{S}^{*}}{{\delta t}_{P}^{*}}/\frac{{V}_{P}}{{V}_{S}}$. Since $\frac{{V}_{P}}{{V}_{S}}$ ratios in our study area range from 1.64–1.84^12^, the corresponding $\frac{{Q}_{P}}{{Q}_{S}}$ ratios will be in the ranges of 0.58 to 0.7. These small $\frac{{Q}_{P}}{{Q}_{S}}$ ratios imply that scattering attenuation is the dominant mechanism of attenuation; as for viscous materials, like magma mushes, $\frac{{Q}_{P}}{{Q}_{S}}$ will reach 2.2–2.6^16^. This finding also rules out the possibility of a massive magma chamber beneath the Taipei metropolis.

## Discussions and conclusions

**Figure 8:**
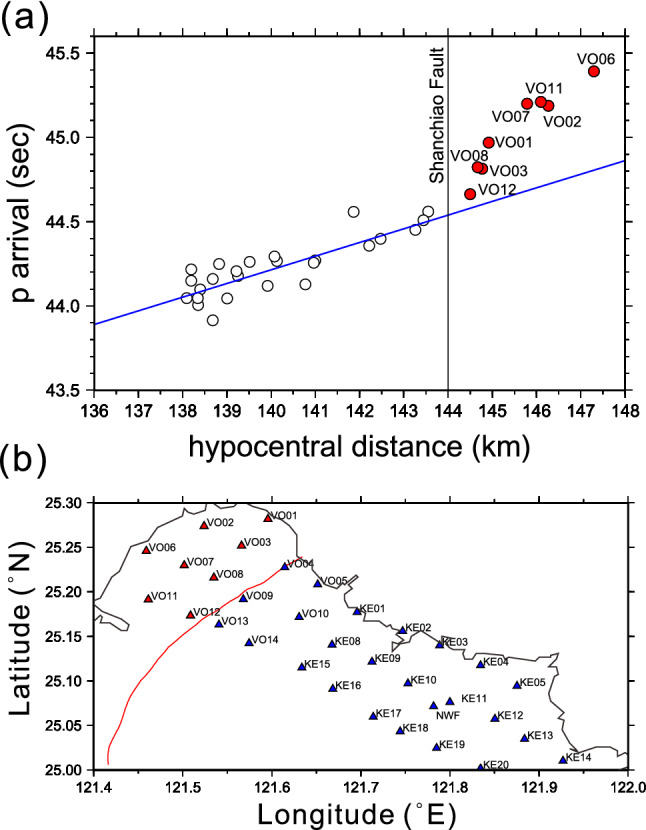
**(a)** The plot of P-wave arrivals of EQ2 against the hypocentral distance. Evident time delays (red dots) occurred when the hypocentral distance exceeds 144 km. The blue line denotes the linear regression from the data set with hypocentral distances less than 144 km. **(b)** Distribution of the seismic stations associated with P-wave lags (the red triangles) and those without P-wave delays (the blue triangles).

We have demonstrated that it is unlikely to have a massive magma chamber beneath the Taipei metropolis. However, there is still a question that remains to be answered: what causes P-wave delays if no massive magma chamber exists? Fig. 8 depicts the relationship between the first P arrival time and the hypocentral distance for EQ2 (the corresponding waveforms are shown in Supplementary Figure S4). As described in the previous study^10^, we also found anomalous P-wave delays when the hypocentral distances exceeded 144 km. It is worth noting that all the stations that recorded the anomalous P-wave delays are on the footwall of the Shanchiao fault (Fig. 8b). More importantly, we observed that the P-wave delays increased with hypocentral distance once the seismic waves passed through the footwall (west side) of the Shanchiao fault regardless of whether they traversed across the postulated magma chamber. This finding excludes that P-wave delays can be served as evidence of the present of a magma reservoir. The most intuitive answer to the P-wave delays is that the seismic wave velocity on the footwall (west) of the Shanchiao fault is smaller than that on the hanging wall^12,21,25^. For example, the ray path from the Shanchiao fault to the station VO06 is 41.3 km for EQ2. Assuming the P wave velocity on the hanging wall is 6 km/s and drops 8% on the footwall, we would arrive at 0.59 s for the time delay of P arrival, which is akin to the observed time lag of 0.6 s (Fig. 8).

According to our study results, we argue that if any magma reservoir presents beneath the Taipei metropolis, the body must be too small (consider that the mean ray interval in Fig. 3 is less than 2 km) to affect the characteristics of seismic waves. A small fraction of magma can explain why volcanic gases contain a tiny amount of helium while exhibiting a high ^3^He/^4^He ratio. The 1367-year-old magnetites found at Shamao Mountain are much younger than the U-Th ages (18–33 Ka) determined from mineral–mineral pairs in the same rock sample^7^. This fact implies that the magnetite may have been generated from the reaction of preexisting volcanic rocks with later acidic, iron-saturated fluids^25^. Such fluids are the typical hot springs found in the TVG. The evidence of the presence of 6 Ka volcanic debris near the summit of Mt. Cising most likely originated from phreatic eruptions^6^. However, the small-scale phreatic eruption does not necessarily indicate water and magma contact at shallow depths. Decompression or high-speed flow of hydrothermal fluid, which can be recharged from crystallization-driven exsolution at depths, may also induce phreatic eruptions^26^. A recent study^21^ showed that a hydrothermal fluid reservoir resides 2 km below the Mt. Cising. The rapid ascent of the hydrothermal fluid through fissures could have triggered subspinodal depressurization and resulted in a weak explosive boiling^27^, which, we believe, is the most likely scenario of the small-scale phreatic eruption found in 6000 BP. Compared with the massive explosion in 2.8–0.1 Ma, the present magma chamber must have been shrinking due to cooling and insufficient magma supply. Without sufficient magma supply, the TVG may stay dormant (except for small-scale phreatic eruptions) or even go extinct if the size of the magma chamber is below a critical one^28^.

## Supplementary Information

Supplementary Information.

## Data Availability

The waveform data that support the findings of this study are available from the Taiwan Earthquake Center (for the Formosa Array), the Taiwan Volcano Observatory at Tatun (for the TVO Array), the Central Weather Bureau of Taiwan (for the CWB Array), the National Center for Research on Earthquake Engineering (for the NCREE Array), and the Industrial Technology Research Institute (for the Ginsan Array), but restrictions apply to the availability of these data, which were used under license for the current study. Data are, however, available from the authors upon reasonable request and with permissions of these organizations.
