# Supplementary material for: Dense seismic arrays deny a massive magma chamber beneath the Taipei metropolis, Taiwan
Source: Sci Rep. 2021 Jan 13;11:1083. doi: 10.1038/s41598-020-80051-4 (PMC7806728; doi:10.1038/s41598-020-80051-4)
Supplement: Supplementary file 1 — Supplementary Information. [file 41598_2020_80051_MOESM1_ESM.docx]

Supplementary Material for

**Dense seismic arrays deny a massive magma chamber beneath the Taipei metropolis, Taiwan**

Y.-L. Yeh^1^, W.-H. Wang^1^, S. Wen^1^

^1^Department of Earth and Environmental Sciences, National Chung Cheng University, Taiwan.

Table S1 Earthquakes employed in this study

| Earthquake | Date(UTC) | Longitude  (°E) | Latitude  (°N) | Focal Depth | Magnitude  (M_L_) |
| --- | --- | --- | --- | --- | --- |
| EQ1 | 1/17/2018 | 121.72 | 25.06 | 140.2 km | 5.5 |
| EQ2 | 3/12/2019 | 121.86 | 25.02 | 138 km | 5.3 |
| EQ3 | 12/16/1015 | 122.13 | 25.22 | 218 km | 4.28 |
| EQ4 | 11/28/2015 | 122.26 | 25.19 | 219 km | 5.15 |
| EQ5 | 12/15/2015 | 122.31 | 25.23 | 217 km | 4.11 |
| EQ6 | 4/13/2015 | 122.40 | 25.29 | 228 km | 4.38 |

(Data provided by the Central Weather Bureau of Taiwan)

Group YC

Group YL-YM

Group YM

Group CT

Group CT-KE

Group KE


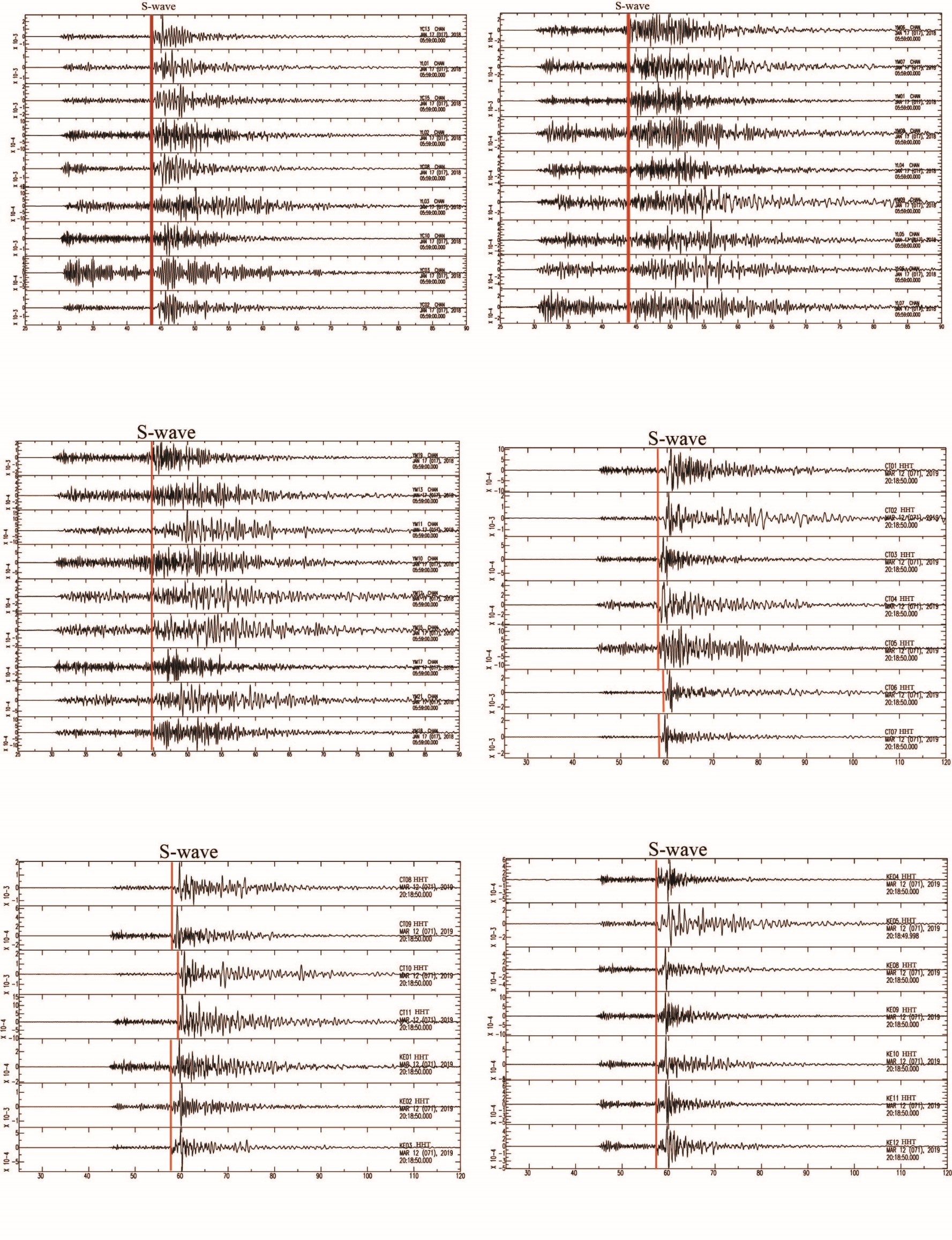


Figure S2. The transverse-component seismograms of EQ1 and EQ2 observed at the seismic stations shown in Fig. 1a. The red lines mark the first S wave arrivals. Groups YC, YL, and YM show the waveforms of the EQ1 observed by the TVO array, and the rest waveforms are the EQ2 observed by the Formosa array. The location of each seismic station is shown in Fig. S3.

Group VO

Group VO

Group KE-PN

Group KE


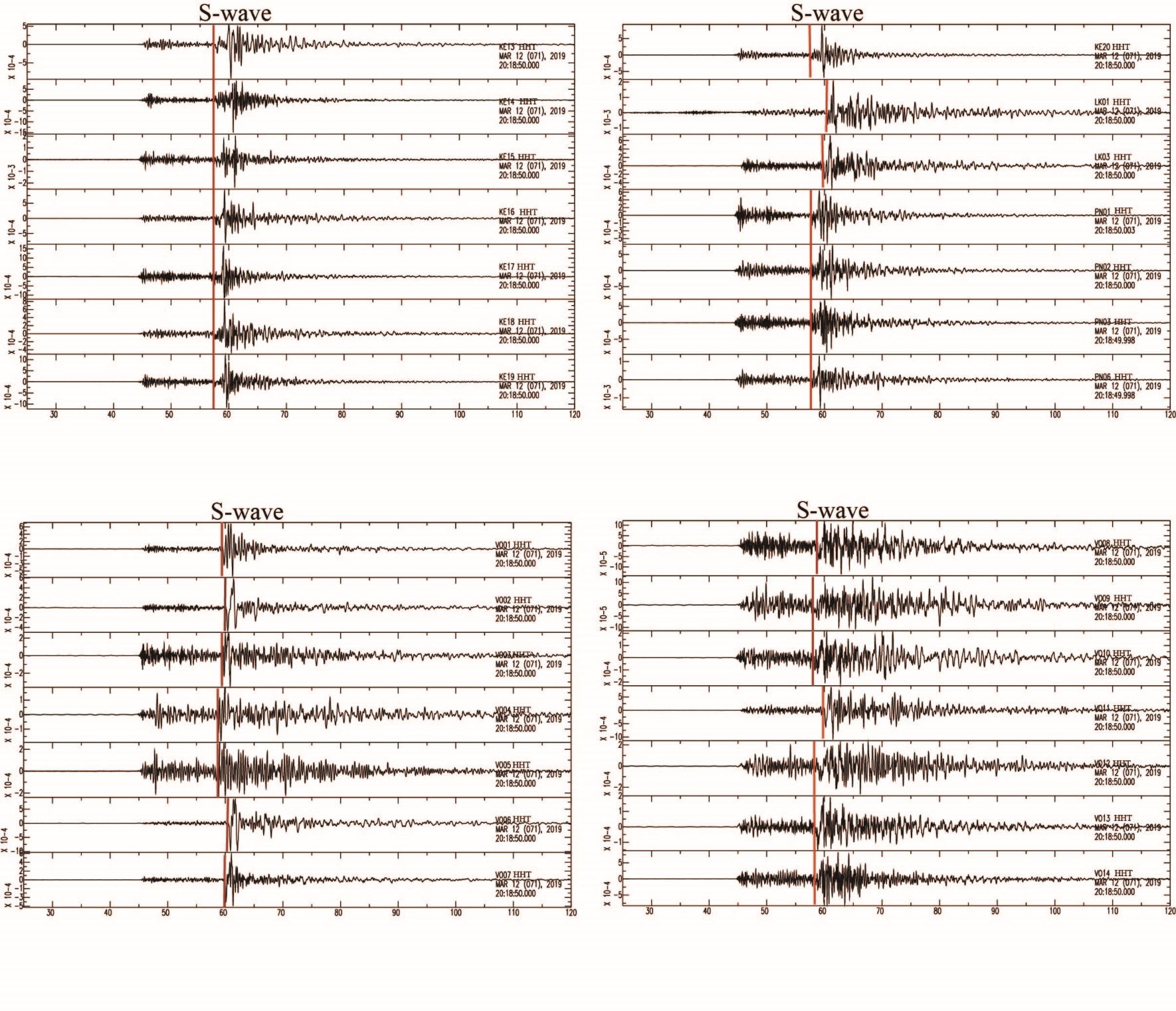


Figure S2. (continued)


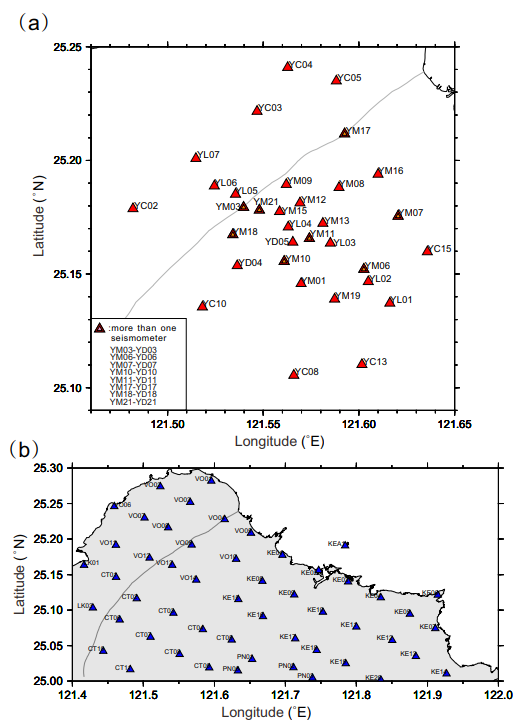


Figure S3. Seismic station locations in the (a) TVO Array and (b) Formosa Array.


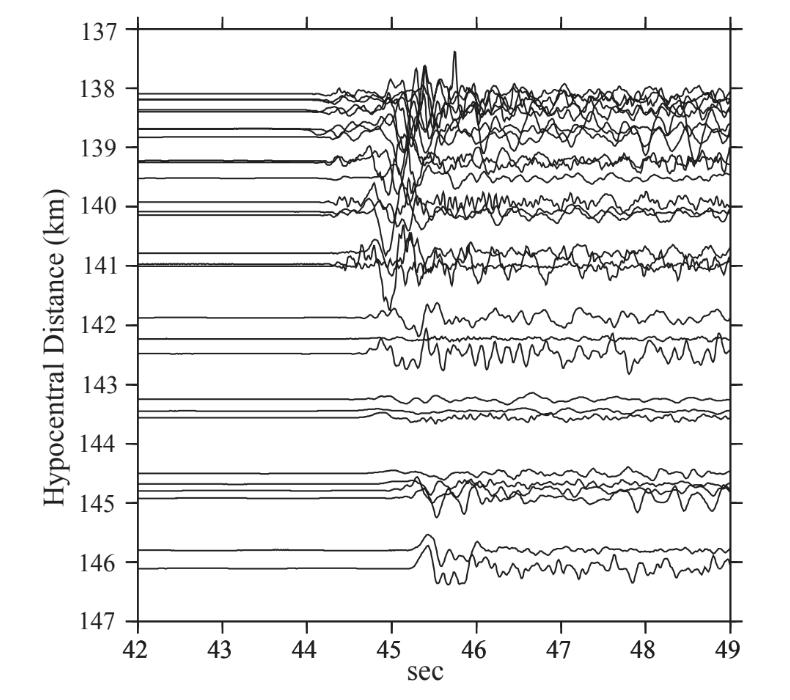


Figure S4. The vertical-component seismograms of the EQ2 aligned with the hypocentral distances.
